# Patterns of acute hospital and specialist palliative care use among people with non-curative upper gastrointestinal cancer

**DOI:** 10.1007/s00520-024-08624-x

**Published:** 2024-06-14

**Authors:** E. G. Boland, K. T. Tay, A. Khamis, F. E. M. Murtagh

**Affiliations:** 1 Hull University Teaching Hospitals NHS Trust, Hull, UK; 2 Wolfson Palliative Care Research Centre, Hull York Medical School, University of Hull, Hull, UK

**Keywords:** Upper gastrointestinal cancer, Non-curative, Specialist palliative care, Hospital admissions, Re-admission 30 days after discharge, Deprivation

## Abstract

**Purpose:**

Upper gastrointestinal (GI) cancers contribute to 16.7% of UK cancer deaths. These patients make high use of acute hospital services, but detail about palliative care use is lacking. We aimed to determine the patterns of use of acute hospital and hospital specialist palliative care services in patients with advanced non-curative upper GI cancer.

**Methods:**

We conducted a service evaluation of hospital use and palliative care for all patients with non-curative upper GI cancer seen in one large hospital, using routinely collected data (2019–2022). We report and characterise hospital admissions and palliative care within the study time period, using descriptive statistics, and multivariable Poisson regression to estimate the unadjusted and adjusted incidence rate ratio of hospital admissions.

**Results:**

The total with non-curative upper GI cancer was 960. 86.7% had at least one hospital admission, with 1239 admissions in total. Patients had a higher risk of admission to hospital if: aged ≤ 65 (IRR for 66–75 years 0.71, IRR 76–85 years 0.68; IRR > 85 years 0.53; *p* < 0.05), or lived in an area of lower socioeconomic status (IMD Deciles 1–5) (IRR 0.90; *p* < 0.05). Over the 4-year period, the rate of re-admission was higher in patients not referred to palliative care (rate 0.52 readmissions/patient versus rate 1.47 readmissions/patient).

**Conclusion:**

People with advanced non-curative gastrointestinal cancer have frequent hospital admissions, especially if younger or from areas of lower socioeconomic status. There is clear association between specialist palliative care referral and reduced risk of hospitalisation. This evidence supports referral to specialist palliative care.

## Introduction

Upper gastrointestinal (GI) cancers are a group of cancers which involve the oesophagus, stomach, small intestine, pancreas, liver, bile duct and gall bladder [1]. Globally, upper GI cancers contribute to 16.6% of new cancer cases and 27.1% of cancer mortality [2]. In the UK, pancreas (2.7%), oesophagus (2.4%), stomach (1.7%) and liver (1.6%) cancers together account for 8.4% of the twenty most common causes of cancer and collectively they contribute to 16.7% of all cancer deaths in the UK  [3]. Although survival rates have increased over the years, upper GI cancers continue to have poor overall prognoses. In a study looking at 40-year trend in overall survival, upper GI cancers (stomach, pancreas and oesophagus) showed little or no improvement in 1, 5 and 10 years survival over this time. The same study reported a 5-year survival of upper GI cancers of between 3.3 and 18.8% [4].

Patients with GI cancers in the last year of life often present with advanced disease and experience high symptom burden [5, 6]. Fatigue, pain, nausea, vomiting and eating-related problems are among some of the predominant physical symptoms reported, in addition to disruption of emotional, social and spiritual functioning [7, 8]. To address these symptoms, interventions such as chemotherapy, radiation therapy and surgical and endoscopic interventions are often used with palliative intent [9, 10]. These interventions show improvement in quality of life [9], but are also associated with increased hospitalizations, and with significant morbidity and mortality [11–13]. Utilization of such interventions in patients with advanced GI cancers appears to be low (12–16%), albeit an increasing trend over time [8, 13]. Sociodemographic and geographical variation are among some of the factors associated with lower utilization of palliative interventions (surgery, chemotherapy, radiation, pain management) in this group of patients [14].

Specialist palliative care services in the hospitals and community in the UK are multidisciplinary teams who provides assessment, advice and support for patients with advanced, progressive and life-limiting disease who have complex physical, psycho-social and spiritual needs [15]. Early integration of specialist palliative care (SPC) has been shown to improve healthcare-related quality of life, patient’s satisfaction and treatment decision making; reduce healthcare utilization and reduce aggressive treatment in patients with advanced cancers [16–19]. In-patients receiving palliative care have higher odds of discharge to hospice care (OR 1.23) and lower odds of ICU admission (OR 0.94) [20, 21]. Thus, early involvement of SPC services is recommended in best practise guidelines [22, 23].

The recent ‘Report of the Lancet Commission on the Value of Death’ highlighted an increase in health care utilization and health spending in patients towards the last year of life, with an exponential increase in the last 30 days [24]. Many of these healthcare utilization are inappropriate and do not benefit patients [24, 25]. In a national audit for end of life care in NHS inpatient providers of acute and community care for all populations in England and Wales, 26% of care provided to patients and 32% of care provided to families/carer were reported to be only fair or poor [26]. A study found that people living in communities with lower socioeconomic status have higher hospital versus home deaths; increased use of acute hospital-based care in the last 3 months of life; reduced odds of using specialist palliative care services in the last year of life, and are consequently more likely to experience worse care [27].

However, little is known about the utilization of inpatient SPC services and acute hospital services in patients with advanced upper GI cancer. Having a better understanding of this can help guide the development of an effective and accessible model of early inpatient SPC service in this group of patients. The aim of this study is to examine the utilization of inpatient SPC services and acute hospital services in patients with advanced upper GI cancer and identify associated factors.

## Methods

### Study design and setting

The UK has a government-sponsored universal healthcare system called the National Health Service (NHS). The NHS consists of a series of publicly funded healthcare systems in the UK. We conducted a service evaluation of hospital and SPC use for all patients with non-curative upper GI cancer seen at Hull University Teaching Hospitals NHS Trust, using secondary analysis of routinely collected hospital data (2019–2022). Two hospitals (total of 1160 beds) in the Trust provide acute care for a local population of approximately 600,000 and provide tertiary services to a regional population of approximately 1.2 million people. The cohort was identified from the Somerset Cancer Register and the National Oesophago-Gastric Cancer Audit (NOGCA) [28, 29]. The Somerset Cancer Register is a web application designed to collect patient data to help healthcare professionals manage the patient’s pathway, from GP referral through to the end of treatment. The cohort identified were patients with newly diagnosed Upper GI cancer and their treatment intent was palliative as decided by the multidisciplinary team. We have followed the UKRI Medical Research Council and UK NHS Health Research Authority guidance to establish this service evaluation and we had appropriate approvals through the Clinical Audit and Effectiveness Team at University Hospital NHS Trust. Consent was not required. This research was conducted in accordance with the Declaration of Helsinki.

### Participants

For the identified cohort, we captured all subsequent hospital admissions (except out-of-area acute hospital use) within the time period, characterising the type, duration and outcome of admission. We selected hospital admissions as our main outcome of interest, as admissions are high in this population, and may not always reflect the optimal place of care for the individual. We decided on 30 days following discharge from any indexed admission as this is a common UK metric when evaluating quality of care the patient received. For those who died between June 2021 and May 2022, we identified admissions in the last year of life.

### Data collection variables

Patient characteristics included age, sex, area-level socio-economic status, cancer site, date of diagnosis, date of death (where applicable), hospitalisations (including duration and route of admission), and if referral to hospital specialist palliative care team occurred. The hospital specialist palliative care team supports patients with complex palliative and terminal care needs in the hospital only and includes both clinical nurse specialists and consultants in palliative medicine.

We also examined patterns of hospital re-admissions within 30 days of discharge among patients with and without referral to the hospital specialist palliative care teams.

We used the Index of Multiple Deprivation to indicate area of socio-economic status, which is the official measure of relative deprivation in England. The domains are combined using the following weights: Income Deprivation, Employment Deprivation, Education, Skills and Training Deprivation, Health Deprivation and Disability, Crime, Barriers to Housing and Services and Living Environment Deprivation. The lower the rank, the lower the socioeconomic status of the area; a decile of 1 means the postcode is in the lowest 10% of the deprivation index, a decile of 10 means the postcode is in the highest 10% [30]. 

### Statistical methods

Statistical analyses were performed using STATA 17. We used descriptive statistics to report the frequencies, means, and standard deviations (SDs), median and interquartile range (IQR) for participants’ characteristics and patterns of hospitalisations. Multivariable Poisson regression was used to estimate the association between characteristics of patients with non-curative upper GI cancers and hospital admissions using an adjusted incidence rate ratio (IRR).

## Results

Total number of patients diagnosed with non-curative upper GI cancer over the 4-year period was 960 (see Table 1 for demographic, diagnostic and survival details).

**Table 1:** Socio-demographics and cancer diagnosis of all non-curative upper GI cancer patients (*N* = 960)

Socio-demographic details	*N*	Proportion
Age		
Mean (SD) in years	Mean 74	(11.2)
Median (IQR) in years	Median 75	(67 – 83)
< 60 years	121	12.6%
≥ 60 years	839	87.4%
Missing	*0*	*0.0%*
Sex		
Men	580	60.4%
Women	380	39.6%
Missing	0	0.0%
Ethnicity		
White British	751	78.2%
Other	24	2.5%
Unknown	185	19.3%
Survival		
Months from diagnosis till death for those who died (*N* = 828)	Mean 6 Median 3	(SD 7) (IQR 1 – 8)
Site of cancer		
Upper GI cancer	960	100%
Pancreas	293	30.5%
Oesophagus	210	21.8%
Gastric	143	14.9%
Unknown primary upper GI site	107	11.2%
Bile duct	88	9.2%
Hepatocellular	83	8.7%
Gallbladder	27	2.8%
Small intestine	9	0.9%
Index of Multiple Deprivation (decile)	*N* = 960	%
1	204	21.2%
2	47	4.9%
3	64	6.7%
4	125	13.0%
5	61	6.4%
6	88	9.2%
7	89	9.2%
8	96	10.0%
9	73	7.6%
10	108	11.3%
Missing	5	0.5%

### Hospital admissions

Over the 4-year period, 832/960 (86.7%) had at least one hospital admission, with 1239 admissions in total. Three hundred fifty-five (37%) patients had 1 admission, 221 (23%) patients had 2 admissions, 123 (12.8%) patients had 3 admissions and 133 (13.9%) patients had ≥ 4 admissions.

For those patients admitted to the hospital (*N* = 832), there were overall 392 (47%) patients that had a hospital specialist palliative care team referral. Two hundred twenty-nine patients (27.5%) had a referral to the hospital specialist palliative care team during their 1st admission. The average length of stay (LOS) was median 10 days (IQR 6 – 16); the mean was 12.6 days (SD 9.4). There were 76 (9.1%) patients who had an ICU stay, with 80 admissions in total and a median LOS of 15 (IQR 10 – 33) days.

In terms of outcomes at the end of period of study, 132 (13.7%) patients were still alive at the end of the data collection period, and 828 (86.3%) had died. For those who died, the median months from diagnosis to death was 4 (IQR 2 – 9), mean months was 7.1 (SD 8.1). The number of days from referral to hospital specialist palliative care team to death was a median of 18 days (IQR 7 – 50), and the mean was 46 days (SD 90).

### Hospital re-admissions

Over the 4-year period, there were 420 admissions among 250 patients re-admitted within 30 days of discharge. Table 2 shows the number of patients that have been admitted and re-admitted to hospital.

**Table 2:** Number of patients admitted to the hospital and the number of patients re-admitted including total admissions and readmissions

All patients included in the study	*N* = 960
Patients admitted to the hospital at least once	832 (86.7%)
Average admissions per patient	
Median (IQR)	1 (1 – 3)
Mean ± SD	1.9 ± 1.6
Min–Max	0 – 14
Total admissions	1239
Patients admitted to the hospital at least once	*N* = 832
Patients readmitted to the hospital after 1st admission	477 (57.3%)
Average readmissions per patient	
Median (IQR)	2 (1 – 3)
Mean ± SD	2.1 ± 1.5
Min – Max	1 – 13
Total re-admissions	1004
Patients readmitted within 30 days	250 (30.1%)
Average readmissions within 30 days per patient	
Median (IQR)	1 (1 – 2)
Mean ± SD	1.7 ± 1.4
Min–Max	1 – 10
Total re-admissions within 30 days	420

Among the patients admitted at least once, we examined patterns of hospital re-admission in relation to hospital specialist palliative care referral. Over the 4-year period, there were 120 re-admissions among 229 patients previously referred to hospital specialist palliative care (rate 0.52 readmissions/patient/4 years) versus 884 re-admissions among 603 patients not previously referred to hospital specialist palliative care (rate 1.47 readmissions/patient/4 years).

Using Poisson regression analysis (Table 3), we looked at hospital admissions and found a significant association that the older the patients were, the less likely they were admitted to the hospital compared to patients aged ≤ 65. (IRR for 66 – 75 years 0.71, IRR 76 – 85 years 0.68; IRR > 85 years 0.53; *p* < 0.05). Patients living in areas of lower socioeconomic status (deprivation index 1–5) were more likely to be admitted to hospital compared to patients living in areas with less deprivation (deprivation index 6 – 10) (IRR 0.90; *p* < 0.05). Non-British patients were more likely to be admitted to hospital compared to British people (IRR 0.89; *p* < 0.05). Patients diagnosed with cancer of unknown primary were less likely to be admitted to the hospital compared to patients with bile duct, gall bladder, liver and small intestine cancers (IRR 0.69; *p* < 0.05).

**Table 3:** Poisson regression analysis showing adjusted and unadjusted incidence rate ratios (IRR) and respective 95% confidence intervals (95% CI) in the analysis of the factors associated with number of admissions

	Unadjusted IRR (95% CI)	Adjusted IRR (95% CI)	*p* value
Outcome: number of admissions	*N* = 960	*N* = 955 Pseudo R2 = 0.0352	
Age			
≤ 65	1	1	
66 – 75	0.74 (0.66 – 0.83)*	0.71 (0.63 – 0.80)*	** < 0.001**
76 – 85	0.69 (0.61 – 0.78)*	0.68 (0.60 – 0.77)*	** < 0.001**
> 85	0.55 (0.47 – 0.65)*	0.53 (0.45 – 0.63)*	** < 0.001**
Gender			
Female	1	1	
Male	0.96 (0.88 – 1.06)	0.93 (0.85 – 1.03)	0.155
Ethnicity			
British	1	1	
Others	0.92 (0.82 – 1.03)	0.89 (0.79 – 1.00)*	**0.049**
Diagnosis			
Bile duct, gallbladder, liver, small intestine	1	1	
Oesophageal	0.91 (0.80 – 1.05)	0.88 (0.77 – 1.01)	0.071
Pancreatic	0.94 (0.83 – 1.06)	0.90 (0.80 – 1.03)	0.119
Stomach	0.99 (0.86 – 1.15)	0.96 (0.83 – 1.12)	0.636
Unknown primary	0.70 (0.58 – 0.84)*	0.69 (0.57 – 0.83)*	** < 0.001**
Outcome			
Alive	1	1	
Died	1.35 (1.16 – 1.57)*	1.43 (1.23 – 1.67)*	** < 0.001**
IMD deciles	*N* = 955		
1 – 5	1	1	
6 – 10	0.86 (0.79 – 0.95)*	0.90 (0.82 – 0.99)*	**0.028**

*Significant *p* < 0.05

We further analysed re-admissions to the hospital over the 4-year period (Table 4). The older the patients were, the less likely they were admitted to the hospital compared to patients aged ≤ 65 (IRR for 66 – 75 years 0.57, IRR 76 – 85 years 0.55; IRR > 85 years 0.37; *p* < 0.05). Interestingly, patients who were previously referred to the hospital specialist palliative care team at their first admission had a 64% less chance of being readmitted compared to patients who were not referred (IRR 0.36; *p* < 0.05).

**Table 4:** Poisson regression analysis showing adjusted and unadjusted Incidence rate ratios (IRR) and respective 95% confidence intervals (95% CI) in the analysis of the factors associated with number of total re-admissions

	Unadjusted IRR (95% CI)	Adjusted IRR (95% CI)	*p* value
Outcome: number of total re-admissions	*N* = 832	*N* = 827 Pseudo R2 = 0.1030	
Age			
≤ 65	1	1	
66 – 75	0.60 (0.51 – 0.70)*	0.57 (0.49 – 0.67)*	** < 0.001**
76 – 85	0.57 (0.49 – 0.67)*	0.55 (0.47 – 0.65)*	** < 0.001**
> 85	0.36 (0.28 – 0.45)*	0.37 (0.29 – 0.47)*	** < 0.001**
Gender			
Female	1	1	
Male	1.03 (0.91 – 1.17)	0.96 (0.84 – 1.09)	0.535
Ethnicity			
British	1	1	
Others	0.93 (0.80 – 1.09)	0.87 (0.74 – 1.02)	0.080
Diagnosis			
Bile duct, gallbladder, liver, small intestine	1	1	
Oesophageal	0.92 (0.76 – 1.10)	0.80 (0.67 – 0.97)*	**0.021**
Pancreatic	0.89 (0.75 – 1.05)	0.90 (0.76 – 1.06)	0.202
Stomach	0.98 (0.80 – 1.19)	0.92 (0.75 – 1.12)	0.388
Unknown primary	0.50 (0.38 – 0.66)*	0.57 (0.43 – 0.76)*	** < 0.001**
Palliative Care request after 1st admission			
No	1	1	
Yes	0.36 (0.30 – 0.43)*	0.36 (0.29 – 0.43)*	** < 0.001**
Outcome			
Alive	1	1	
Died	1.23 (1.0 – 1.52)*	1.68 (1.36 – 2.08)*	** < 0.001**
IMD deciles	*N* = 527		
1 – 5	1	1	
6 – 10	0.87 (0.76 – 0.98)*	0.90 (0.79 – 1.02)	0.108

*Significant *p* < 0.05

Lastly, we looked at re-admissions to the hospital within 30 days of discharge from the hospital (Table 5). The older the patients were, the less likely they were admitted to the hospital compared to patients aged ≤ 65. (IRR for 66 – 75 years 0.39, IRR 76 – 85 years 0.34; IRR > 85 years 0.16; *p* < 0.05), patients living in areas of lower socioeconomic status (deprivation index 1 – 5) were more likely to be admitted to hospital compared to patients living in areas with less deprivation (deprivation index 6 – 10) (IRR 0.77; *p* < 0.05). Patients with stomach cancer had a 24% increased risk of re-admission within 30 days of discharge compared to cancers (liver, bile duct, gallbladder and small intestine). Interestingly, patients who were referred to the hospital specialist palliative care team after their first admission were less likely to be readmitted compared to patients who were not referred (IRR 0.77; *p* < 0.05).

**Table 5:** Poisson regression analysis showing adjusted and unadjusted Incidence rate ratios (IRR) and respective 95% confidence intervals (95% CI) in the analysis of the factors associated with number of re-admissions within 30 days of discharge

	Unadjusted IRR (95% CI)	Adjusted IRR (95% CI)	*p* value
Outcome: Number of readmissions within 30 days	*N* = 832	*N* = 827 Pseudo R2 = 0.0909	
Age			
≤ 65	1	1	
66 – 75	0.42 (0.33 – 0.53)*	0.39 (0.31 – 0.49)*	** < 0.001**
76 – 85	0.35 (0.27 – 0.45)*	0.34 (0.27 – 0.44)*	** < 0.001**
> 85	0.17 (0.11 – 0.27)*	0.16 (0.10 – 0.25)*	** < 0.001**
Gender			
Female	1	1	
Male	0.95 (0.78 – 1.15)	0.86 (0.70 – 1.05)	0.132
Ethnicity			
British	1	1	
Others	0.99 (0.78 – 1.25)	0.89 (0.70 – 1.13)	0.323
Diagnosis			
Bile duct, gallbladder, liver, small intestine	1	1	
Oesophageal	1.05 (0.78 – 1.42)*	0.90 (0.66 – 1.23)	0.513
Pancreatic	1.15 (0.88 – 1.5)	1.03 (0.78 – 1.35)	0.847
Stomach	1.39 (1.02 – 1.88)*	1.24 (0.91 – 1.69)*	0.166
Unknown primary	0.75 (0.50 – 1.13)	0.78 (0.52 – 1.17)	0.224
Palliative Care request after 1st admission			
No	1	1	
Yes	0.78 (0.62 – 0.98)*	0.77 (0.61 – 0.98)*	**0.031**
Outcome			
Alive	1	1	
Died	1.66 (1.15 – 2.39)*	2.09 (1.44 – 3.03)*	** < 0.001**
IMD deciles	N = 827		
1 – 5	1	1	
6 – 10	0.75 (0.62 – 0.91)*	0.81 (0.67 – 0.99)*	**0.041**

*Significant *p* < 0.05

### Last year of life

Between June 2021 and May 2022, 257 patients died. The mean survival in this group from diagnosis to death was 7 months, (SD 8), and the median was 3 months (range 0 – 29). 231/257 (89.9%) patients had at least one hospital admission in the last year of their life. 82/257 (31.9%) had one hospital admission, 149 (58%) had more 2 or more hospital admission, with 562 admissions in total. 345/562 (61.4%) admissions were unplanned via ED, 91/562 (16.2%) were unplanned not via ED, and 126/562 (22.4%) were elective.

From their last hospital admission (*N* = 231), the median number of months until death was 1 (IQR 1 – 3), and the mean number of months was 2.9 (SD 4.9).

Out of those patients who had a hospital admission, 127/231 patients (55%) had referrals to the hospital specialist palliative care teams in the last year of life.

There were 149 patients re-admitted to the hospital out of the 231 patients. Within 30 days of discharge, there were 42 patients re-admitted with a median LOS of 8 days.

For last year of life only, among the 231 patients admitted, there were 38 re-admissions among 61 patients referred to hospital specialist palliative care (rate 0.62 readmissions/patient/year) versus 293 re-admissions among 170 patients not referred to hospital specialist palliative care (rate 1.72 readmissions/patient/year).

## Discussion

This analysis identified that younger patients and those from areas of lower socioeconomic status have more hospital admissions. Hospital specialist palliative care referral was found to markedly reduce subsequent unplanned admissions, adjusting for age, gender, ethnicity, diagnosis and IMD deciles.

A study looking at hospital use at the end of life reported that patients had an average of 2.3 admissions and 30 bed days for every person that died in the past 12 months of life; and younger patients had higher admission rates in the last year of life [31]. A report about emergency admissions in the 3 months before death showed that over 25% of patients had cancer as the underlying cause of death, and three-quarters spent at least 13 days in hospital, most were aged 70 and older, and the percentage of admission was lower in White British than all other ethnic groups and was higher in those patients living areas of lower socioeconomic status [32].

Although studies are published reporting early referrals to palliative care help symptom burden, quality of life and decrease aggressive end of life treatments, there is still no clear guidance on when is the best time to refer to specialist palliative care teams especially in a hospital setting [33–36]. A study looked at the effects of palliative care service delivery in routine care showed that patients were less likely to die in hospital and avoided emergency hospital admissions within the last 4 weeks of life if they had a longer interval between the first contact with a palliative care team (whether hospital or community) and death [37]. In our data, patients in their last year of life showed that 55% had referrals to the hospital specialist palliative care teams and the median number of days from referral to the specialist palliative care team to death was 21 days. Interestingly in our study, patients referred to hospital specialist palliative care were notably less likely to be re-admitted, although may be closer to death. A prospective study collected self-reported symptoms of hospitalised patients with advanced cancer (32% had GI cancer) and looked at the relationship between symptoms and hospital utilization, the authors reported that these patients experienced high rates of uncontrolled physical and psychological symptoms and were significantly associated with prolonged hospitalizations and a higher risk for unplanned hospital re-admissions [38].

The incidence and mortality from cancer is increasing worldwide, with an estimated 47% increase in 2040 assuming the national rates remain similar [2]. Patients with gastrointestinal cancer having complex problems especially towards the end of life. A study of patients with gastrointestinal cancer in the last 30 days of life from Canada showed high rates of hospital utilisation, 45.9% of patients attended ED, 59.3% had a hospital admission, 6.3% had an ICU admission and 44.6% died in hospital [39]. A retrospective series of consecutive hospital admissions in patients with GI cancer identified 19% of hospitalizations as potentially avoidable and these were associated with poor performance status, refractory metastatic cancer, oncologists advising hospice care, age 70 and over and three or more hospital admissions over the preceding year; survival in these patients with potentially avoidable hospitalisations was markedly decreased [11]. Another study looked at patients with advanced cancer receiving outpatient palliative care who presented to ED, where 23% of visits where potentially avoidable and they reported that non-white ethnicity was another independent predictor for presentation to ED [40]. In our 4-year study, there were 1700 ED attendances and 62.5% were admitted to hospital.

There are several limitations to this study. This is a retrospective data analysis, therefore the reasons for ED visits and hospital admissions were not known and might have been unavoidable. Patients might have had a preference for hospital admission. Also, we do not know if the patients were known to an outpatient or community palliative care team or the hospice. The study included patients who had presented to a university teaching hospital in Northern England that covers a large geographical area however this study might not be applicable to the general population of cancer patients. Also, data was obtained from a live hospital system and thus likely to represent omissions and errors within the system. Another important factor is that this study occurred during the COVID pandemic, where most notably, between 2019 and 2022, the total number of hospital admissions in England fell [41] and this might have skewed the acute hospital admission rate.

## Conclusion

In conclusion, our novel findings demonstrate that people with advanced non-curative gastrointestinal cancer have frequent hospital admissions, especially if younger or from more areas of lower socioeconomic status. However, they have less likelihood of hospital re-admissions if the patients were referred to the hospital specialist palliative care team. Further work is needed to understand how best to support these group of patients with short prognosis and provide optimal out-patient care or community care. It is also important to understand the variation of care needed for these different patient groups in order to target scarce health resources to best support those with advanced illness.

## Data Availability

No datasets were generated or analysed during the current study.
